# Spironolactone-Induced Lichenoid Drug Reaction and Subsequent Diffuse Eruptive Squamous Cell Carcinomas Successfully Treated With Systemic Methotrexate

**DOI:** 10.7759/cureus.17713

**Published:** 2021-09-04

**Authors:** Anuj Kunadia, Kenneth Shulman, Naveed Sami

**Affiliations:** 1 Medicine, University of Central Florida College of Medicine, Orlando, USA; 2 Dermatopathology, New York Medical College, Valhalla, USA; 3 Internal Medicine, University of Central Florida College of Medicine, Orlando, USA

**Keywords:** keratoacanthoma, lichenoid drug eruption, spironolactone, spironolactone drug reaction, cutaneous drug reaction, squamous cell carcinoma

## Abstract

Antihypertensive agents such as spironolactone have been reported to cause lichenoid drug eruptions. Eruptive keratoacanthomas (KA), considered to be well-differentiated squamous cell carcinoma (SCC), may develop in the setting of such lichenoid reactions. Thus, definitive treatment is imperative. This case report describes a patient on spironolactone who developed a lichenoid drug eruption followed by eruptive KAs and SCC. The treatment approach used systemic methotrexate. While most treatment regimens for widespread eruptive KA/SCC employ intralesional methotrexate, this case demonstrated the utility of its systemic counterpart. This may have also facilitated the resolution of the patient’s lichenoid eruption. There are only three other reports in the literature describing a spironolactone-induced lichenoid drug eruption. Further investigations are needed to evaluate the adverse cutaneous effects of spironolactone as well as the efficacy of systemic methotrexate in treating patients with a significant number of SCCs arising from lichenoid drug eruptions.

## Introduction

Lichenoid drug eruptions have been attributed to various drugs. Antihypertensive agents such as methyldopa and, less frequently, spironolactone have been reported to cause cutaneous eruptions demonstrating clinical and histopathological characteristics consistent with lichen planus [1]. Additionally, there have been reports of eruptive keratoacanthoma (KA) developing in the setting of such lichenoid reactions [2]. KAs are considered to be well-differentiated squamous cell carcinoma (SCC) with metastatic potential. Thus, definitive treatment is imperative to avoid potentially life-threatening complications [3,4]. This case describes a patient on spironolactone who presented with an associated lichenoid drug eruption followed by eruptive KAs and SCC, treated with systemic methotrexate.

## Case presentation

**Figure 1:**
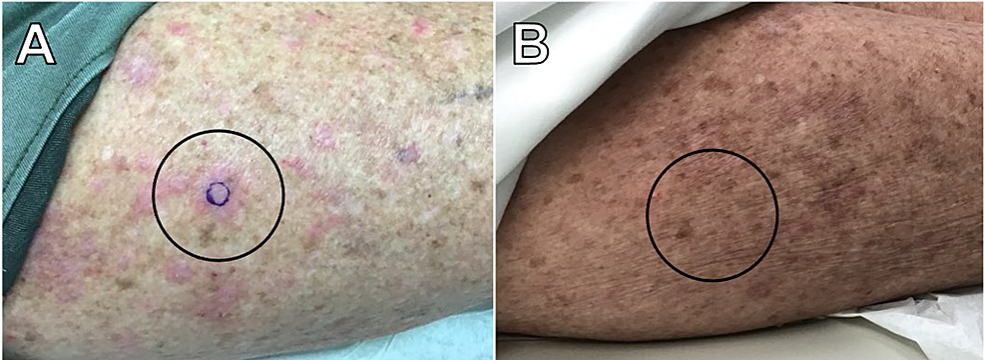
A. Erythematous irregularly shaped macules and plaques on the right lateral thigh (before treatment). B. Resolved lesions on the right lateral thigh (after treatment) with mild residual erythema.

**Figure 2:**
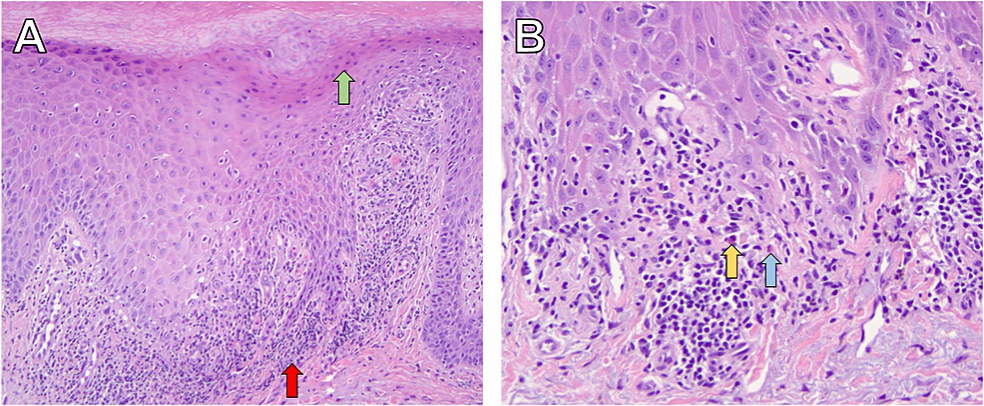
A. Biopsy demonstrating lichenoid dermatitis (red arrow) with focal parakeratosis (green arrow) at 10x magnification. B. Biopsy demonstrating lichenoid with interface dermatitis (blue arrow) changes and a few eosinophils dermatitis (yellow arrow) at 20x magnification.

A 74-year-old female presented with a six-month history of a diffuse pruritic cutaneous eruption on the upper and lower extremities (images of lower extremity are shown in Figure 1 (A, B). The eruption started three weeks after starting spironolactone (50mg/d) for the treatment of hypertension. Her past medical history included polymyalgia rheumatica (PMR) managed with prednisone (3mg/d). The patient did not have any other associated history including infectious hepatitis (B or C) or thyroid dysfunction. A punch biopsy of the skin lesion revealed a psoriasiform and lichenoid dermatitis with eosinophils most consistent with a drug eruption (Figure 2). Spironolactone was considered the most likely cause due to the timeline of its initiation and onset of the rash. The patient was treated with topical triamcinolone 0.1% ointment and tacrolimus 0.1% ointment each applied to affected areas once a day and intramuscular (IM) triamcinolone injection 1 mL of 40.0 mg/mL.

**Figure 3:**
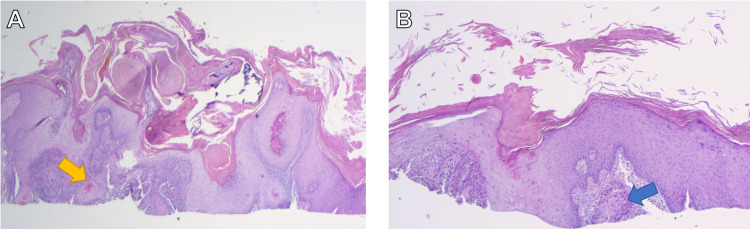
A. Biopsy from the left radial dorsal hand demonstrating a crateriform proliferation of atypical keratinocytes with glassy eosinophlic cytoplasms infiltrating the dermis (yellow arrow) at 2x magnification, consistent with squamous cell carcinoma-keratoacanthoma type. B. Biopsy from the right proximal dorsal forearm demonstrating irregular aggregates of atypical keratinocytes infiltrating the dermis (blue arrow) at 4x magnification, consistent with a well-differentiated squamous cell carcinoma.

At her one-month follow-up, the patient stated she noticed newer lesions that were different from her initial eruption on the upper and lower extremities. These lesions were tender hyperkeratotic erythematous papules, some of which were crateriform and dome-shaped. Biopsies of multiple lesions from three different anatomical sites revealed SCC-KA type and well-differentiated SCCs (Figure 3). Since there were numerous similar small cutaneous lesions along with her history of PMR, which needed a steroid sparing agent, methotrexate was initiated and titrated to 15mg/wk. She demonstrated notable improvement of both her cutaneous and rheumatological conditions. After three months, only two residual SCCs needed surgical excision. The patient continues to do well at her ten-month follow-up with no new lichenoid eruption and SCCs. 

## Discussion

Spironolactone is a nonselective mineralocorticoid receptor antagonist, which can also bind to androgen and progesterone receptors. It is commonly used to treat hypertension and heart failure, with off-label use in acne. Common adverse effects include gynecomastia and hyperkalemia [5]. A less common side effect is various skin rashes [6-8]. A literature search in PubMed resulted in only three other reports describing a spironolactone-induced lichenoid drug eruption (Table 1). These cases were reported in women aged 62-74 years. One study reported a spironolactone dose of 100mg/day. All cases had cutaneous involvement and exhibited resolution of the lichenoid eruption upon withdrawal of spironolactone, taking an average of seven weeks to resolve (ranging from two weeks to about three months) [1,7,8]. One case in the literature exhibited residual erythema and peripheral hyperpigmentation, similar to our patient, who also had mild erythema. Residual changes were not described in the other cases.

**Table 1:** Review of spironolactone-induced lichenoid cutaneous eruptions reported in literature, including current case. SCC: squamous cell carcinoma

Case	Age	Sex	Time from spironolactone initiation to lichenoid eruption	Dose	Resolution upon withdrawal of spironolactone?	Time from spironolactone cessation to eruption resolution
Current	74	F	3 weeks	50mg/day	Yes	3 months with two residual SCCs requiring excision
Downham [1]	62	F	Not Reported	Not Reported	Yes	Within 3 months with residual erythema and peripheral hyperpigmentation remaining
Lark et al [7]	74	F	7 months	100mg/day	Yes	2 weeks
Schön et al [8]	73	F	Not Reported	Not Reported	Yes	>2 months

Cases of similar eruptions may also arise in the setting of Grinspan’s syndrome, described as a triad of lichen planus, diabetes mellitus, and hypertension. It has been hypothesized that the lichenoid eruptions in Grinspan’s syndrome could be drug-induced from anti-hypertensive and anti-diabetic drugs [9]. While lichenoid eruptions have been more commonly associated with mucosal tissues, cutaneous eruption with nifedipine has also been reported. Moreover, malignant transformation of SCC in Grinspan’s syndrome has been only reported in oral lichen planus. This syndrome was unlikely in our patient due to the absence of oral involvement of her lichenoid eruption.

Cutaneous eruptive KAs arising in the setting of lichenoid reactions have also been previously described in the literature. Programmed cell death 1 (PD1) inhibitors such as nivolumab and pembrolizumab have been reported to induce lichenoid dermatitis and subsequent eruptive KAs [2,10]. While the exact mechanism is unclear, the proliferation of KAs may be analogous to those arising in hypertrophic lichens planus, verrucous lupus erythematosus, and other trauma, during which time wound healing is associated with a lichenoid host response [11]. The inflammatory environment of lichenoid reactions may also facilitate a kerato-acanthomatous reaction in predisposed individuals [2].

There are multiple treatment modalities, both medical and surgical, that have been used in the treatment of KAs including intralesional methotrexate, which may additionally be used for the treatment of SCC [12]. This case demonstrates that this treatment can be extrapolated to the use of systemic methotrexate for treating widespread eruptive KAs/SCCs. Systemic methotrexate may have also facilitated the resolution of the initial lichenoid eruption.

## Conclusions

This case highlights the potential for spironolactone to induce a lichenoid drug reaction and consequent eruption of KAs and SCC. The described approach to treating the patient was to employ systemic methotrexate as an 'umbrella' therapy to successfully treat the widespread cutaneous eruptions along with her PMR as a steroid-sparing agent. Further investigations are needed to evaluate the adverse cutaneous effects of spironolactone, as well as the efficacy of systemic methotrexate in treating patients with a significant number of SCCs arising from lichenoid drug eruptions.
